# Gastrointestinal involvement in Ehlers–Danlos syndrome classical-like type 2 associated with a novel *AEBP1* splice-site variant

**DOI:** 10.1038/s10038-026-01480-z

**Published:** 2026-05-26

**Authors:** Hikaru Nakahara, Tomomi Yamaguchi, Hiroaki Niitsu, Akiko Abe, Ryohei Hayashi, Shiro Oka, Koji Arihiro, Tomoki Kosho, Takao Hinoi

**Affiliations:** 1 Department of Clinical and Molecular Genetics, Hiroshima University Hospital, Hiroshima, Japan; 2 Center for Medical Genetics, Shinshu University Hospital, Matsumoto, Japan; 3 Department of Medical Genetics, Shinshu University School of Medicine, Matsumoto, Japan; 4 Division of Clinical Sequencing, Shinshu University School of Medicine, Matsumoto, Japan; 5 Department of Nursing, Hiroshima University Hospital, Hiroshima, Japan; 6 Department of Gastroenterology, Graduate School of Biomedical and Health Sciences, Hiroshima University, Hiroshima, Japan; 7 Department of Endoscopy, Hiroshima Prefectural Hospital, Hiroshima, Japan; 8 Department of Anatomical Pathology, Hiroshima University Hospital, Hiroshima, Japan; 9 Research Center for Supports to Advanced Science, Shinshu University, Matsumoto, Japan; 10 BioBank Shinshu, Shinshu University Hospital, Matsumoto, Japan; 11 Genomic Medicine Center, Hiroshima University Hospital, Hiroshima, Japan

**Keywords:** Medical genetics, Disease genetics, Gastrointestinal diseases, Genetics research

## Abstract

Ehlers-Danlos syndrome classical-like type 2 (clEDS2) is a rare autosomal recessive connective tissue disorder caused by biallelic loss-of-function variants in the gene encoding adipocyte enhancer-binding protein 1 (*AEBP1*). While cutaneous and skeletal manifestations are commonly observed, gastrointestinal complications, including bowel rupture, have been reported only rarely, and their histopathological basis remains poorly characterized. Here, we report findings of molecular investigations, gastrointestinal histopathological evaluation, and long-term clinical follow-up in the 16th reported patient with *AEBP1*-related clEDS2, complicated by spontaneous bowel perforation. A homozygous *AEBP1* splice-site variant (NM_001129.5:c.1401-2 A > G) was identified by a custom next-generation sequencing–based panel analysis for hereditary connective tissue disorders. Transcript-level analysis by reverse transcription-polymerase chain reaction and Sanger sequencing demonstrated complete skipping of exon 12, resulting in a frameshift and predicted loss of function. Clinically, the patient experienced postoperative perforation in the sigmoid colon shortly after rectal cancer surgery, followed nearly 20 years later by spontaneous small intestinal perforation. Histopathological examination of the affected colonic tissue demonstrated mildly widened intermuscular spaces without other overt structural abnormalities. Notably, repeated upper and lower gastrointestinal endoscopic procedures were performed during long-term oncological surveillance without procedure-related bowel perforation. This observation could offer an opportunity to learn about gastrointestinal involvement in *AEBP1*-related clEDS2 and suggests that its gastrointestinal manifestations may differ in clinical context from those typically associated with vascular Ehlers-Danlos syndrome, warranting further investigation as additional cases are accumulated.

## Introduction

Ehlers-Danlos syndrome (EDS) is a heterogeneous group of hereditary connective tissue disorders characterized by joint hypermobility, skin hyperextensibility, and tissue fragility [1, 2]. To date, 14 subtypes of EDS have been recognized, and pathogenic variants in genes involved in collagen biosynthesis and extracellular matrix homeostasis have been identified in 13 of these subtypes [1, 3]. Among the 14 subtypes, EDS classical-like type 2 (clEDS2) was identified most recently, which was first described in 2016 [4]. Currently, 15 affected individuals from 13 families have been reported as clEDS2 [5–13]. Although cutaneous and skeletal manifestations are commonly observed, the reported clinical spectrum also includes infrequent but potentially life-threatening complications, such as atrial or aortic aneurysms or dissections, spontaneous pneumothorax, and bowel rupture, each documented in only one or two individuals [2, 12]. Owing to the limited number of reported cases, detailed molecular, pathological, and longitudinal clinical data remain scarce.

clEDS2 is caused by biallelic loss-of-function variants in the gene encoding adipocyte enhancer-binding protein 1 (*AEBP1*), which plays a critical role in collagen polymerization and extracellular matrix organization [5]. AEBP1 is a multidomain protein containing thrombospondin repeats, a collagen-binding discoidin domain, and a catalytically inactive metallocarboxypeptidase domain [14]. To date, 18 distinct pathogenic variants have been reported across these functional domains [13], indicating that pathogenic variants are widely distributed throughout *AEBP1*. However, the relationship between specific molecular alterations and clinical manifestations, particularly gastrointestinal complications, remains incompletely understood.

Among the gastrointestinal complications reported in *AEBP1*-related clEDS2, spontaneous bowel rupture has been documented in one previously reported individual [5, 12]. However, detailed histopathological evaluation of the gastrointestinal tract in such cases has not been systematically described, leaving the structural basis of gastrointestinal fragility largely unexplored. In this context, this 16th reported case of *AEBP1*-related clEDS2, complicated by spontaneous bowel perforation and a newly identified *AEBP1* variant, could offer a rare opportunity to learn about gastrointestinal involvement in this disorder. Accordingly, findings of molecular investigations, gastrointestinal histopathology, and longitudinal clinical follow-up are described and evaluated in this case.

## Material and methods

### Patient and clinical phenotyping

This study was approved by the Ethical Committee for Epidemiology of Hiroshima University (Approval number; E2024-0153-01) and the Ethics Committee of Shinshu University School of Medicine (Approval number; 6070); and informed consent was obtained from the patient. All analyses were performed in accordance with the Declaration of Helsinki and relevant guidelines and regulations. Clinical information was obtained from the patient or by reviewing the medical records. Hematoxylin and eosin (H&E) staining and Masson’s trichrome (MT) staining for the colonic specimens were reviewed by the pathologists in Hiroshima University Hospital.

### Next generation sequencing

A next-generation sequencing (NGS) panel-based analysis was performed as previously described [12]. In brief, genomic DNA was extracted from peripheral blood using QIAamp DNA Blood Mini Kit on a QIAcube (Qiagen, Valencia, CA, USA), and 52 genes associated with EDS and other hereditary connective tissue disorders were examined by an Ion AmpliSeq custom panel on an Ion Torrent system (Ion Chef and Ion GeneStudio S5, Thermo Fisher Scientific, Waltham, MA, USA).

### Reverse transcription-polymerase chain reaction and Sanger sequencing

RNA was extracted from the patient’s blood by NucleoSpin RNA Blood (Takara, Kusatsu, Japan), and then, complementary DNA (cDNA) was synthesized with ReverTra Ace qPCR RT Master Mix (TOYOBO, Osaka, Japan). Subsequently, reverse transcription-polymerase chain reaction (RT-PCR) was performed by Quick Taq HS DyeMix (TOYOBO) with 2 different primer sets (Supplementary Table 1 and Fig. 3A). After DNA agarose electrophoresis, affected amplicons were extracted by FastGene Gel/PCR Extraction Kit (Nippon Genetics, Tokyo, Japan) to perform Sanger sequencing with the same primers we used in RT-PCR as an outsourcing service by Eurofins Genomics (Tokyo, Japan).

## Results

### Clinical information

**Fig. 1:**
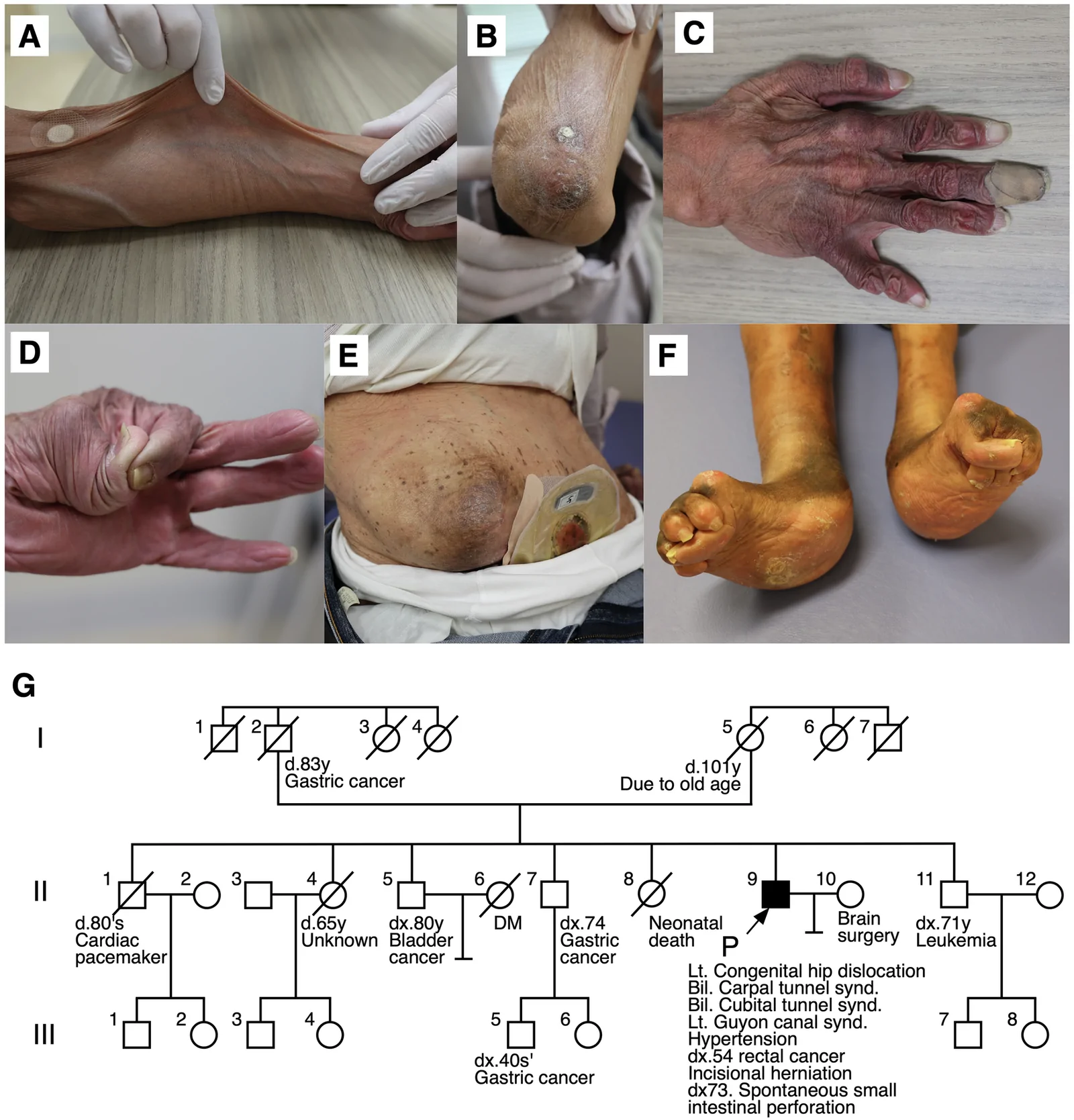
Clinical phenotype of the 16th case of Ehlers-Danlos syndrome classical-like type 2. Physical findings of the patient. At the age of 74, the patient presented with cutaneous hyperelasticity on the forearm (**A**), atrophic scars on the elbow (**B**), progeroid hands (**C**), thumb dislocation (**D**), incisional hernia (**E**), and foot deformities (**F**). **G** Pedigree chart. Of note, there was no obvious parental consanguinity or affected relatives

A 54-year-old Japanese man who was diagnosed with rectal cancer, was referred to the department of general surgery in our hospital. He had a history of left congenital hip dislocation, and also had multiple connective tissue fragility-related symptoms including skin hyperextensibility (Fig. 1A), bruisability, decreased muscle strength in his hand, bilateral carpal tunnel syndrome, bilateral cubital tunnel syndrome, and left Guyon canal syndrome. As a hereditary connective tissue disorder was suspected, he underwent radical proctosigmoidectomy without anastomosis (Hartmann’s procedure). Although the postoperative course was initially uneventful, postoperative perforation occurred in the sigmoid colon just proximal to the colostomy site two months after the surgery, which required diverting ileostomy and abdominal drainage. During the postoperative follow-up, he developed atrophic scars on the elbow, progeroid hands, thumb dislocation, incisional hernia, and foot deformities (Fig. 1B–E). Almost 20 years after the rectal surgery, he experienced spontaneous small intestinal perforation which was conservatively treated by fasting and antibiotics.

There was no obvious parental consanguinity or affected relatives (Fig. 1G). Based on the available family history, no clear shared geographic origin suggestive of a founder effect was identified. After conservative treatment for spontaneous small intestinal perforation, a custom NGS panel-based analysis for hereditary connective tissue disorders was performed, and identified a novel *AEBP1* splice-site variant (NM_001129.5:c.1401-2 A > G) homozygously.

### Molecular investigation of a novel *AEBP1* variant

**Fig. 2:**
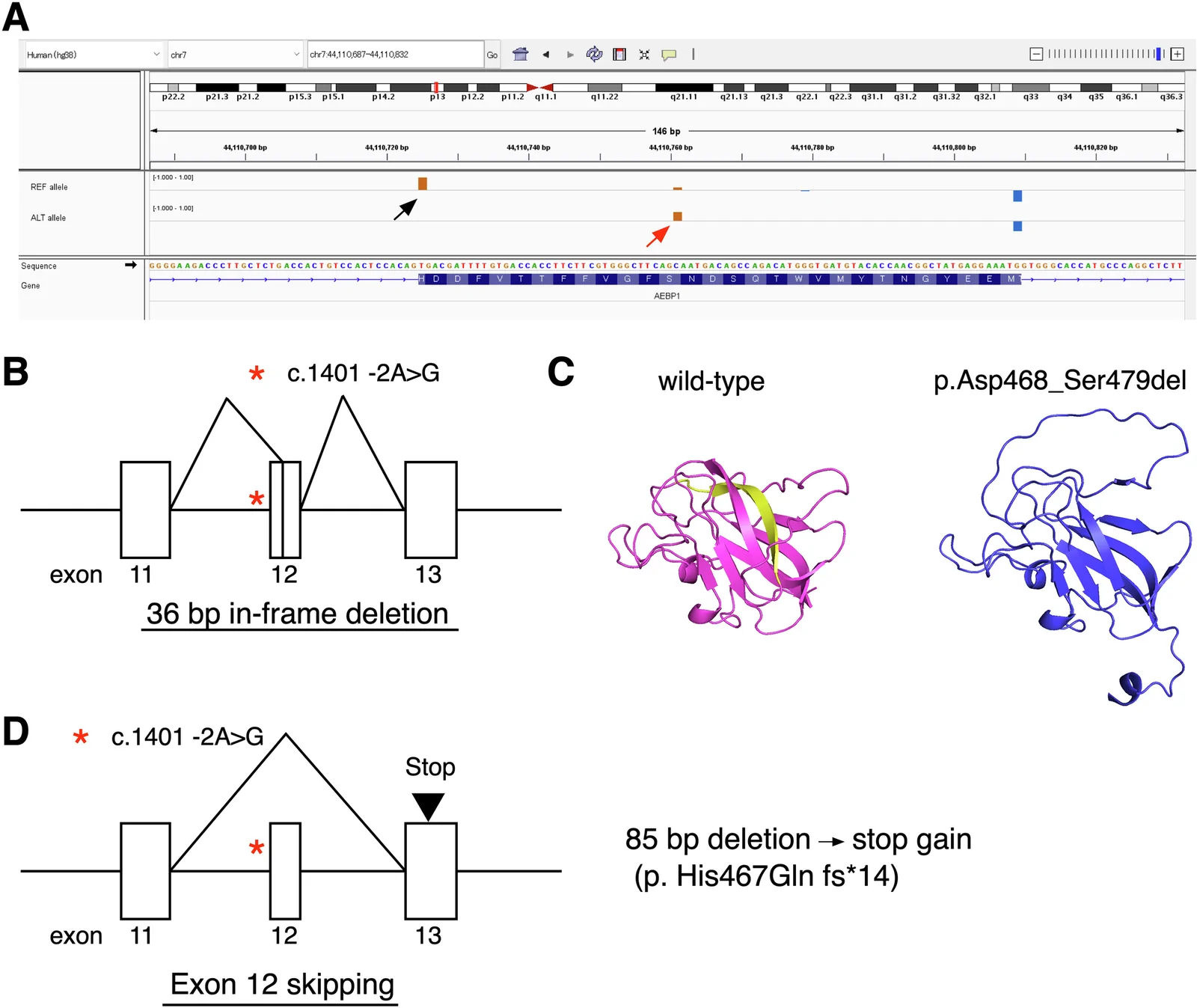
In silico prediction of a novel *AEBP1:*c.1401-2 A > G variant. **A** Prediction of the splicing alteration by SpliceAI. Splice donor and acceptor signals are indicated as orange and blue bars, respectively, which are visualized by Integrative Genomics Viewer (IGV). Arrows indicate alteration of splice acceptor signals between *AEBP1:*c.1401-2 A > G variant and reference sequence. Based on these data, two splice alterations were predicted (**B** and **D**). Prediction 1: 36 bp in-frame deletion (**B**), which may result in three-dimensional conformational change in the discoidin domain of AEBP1 protein (**C**). **D** Prediction 2: Exon 12 skipping, resulting in frameshift and truncation of the protein

**Fig. 3:**
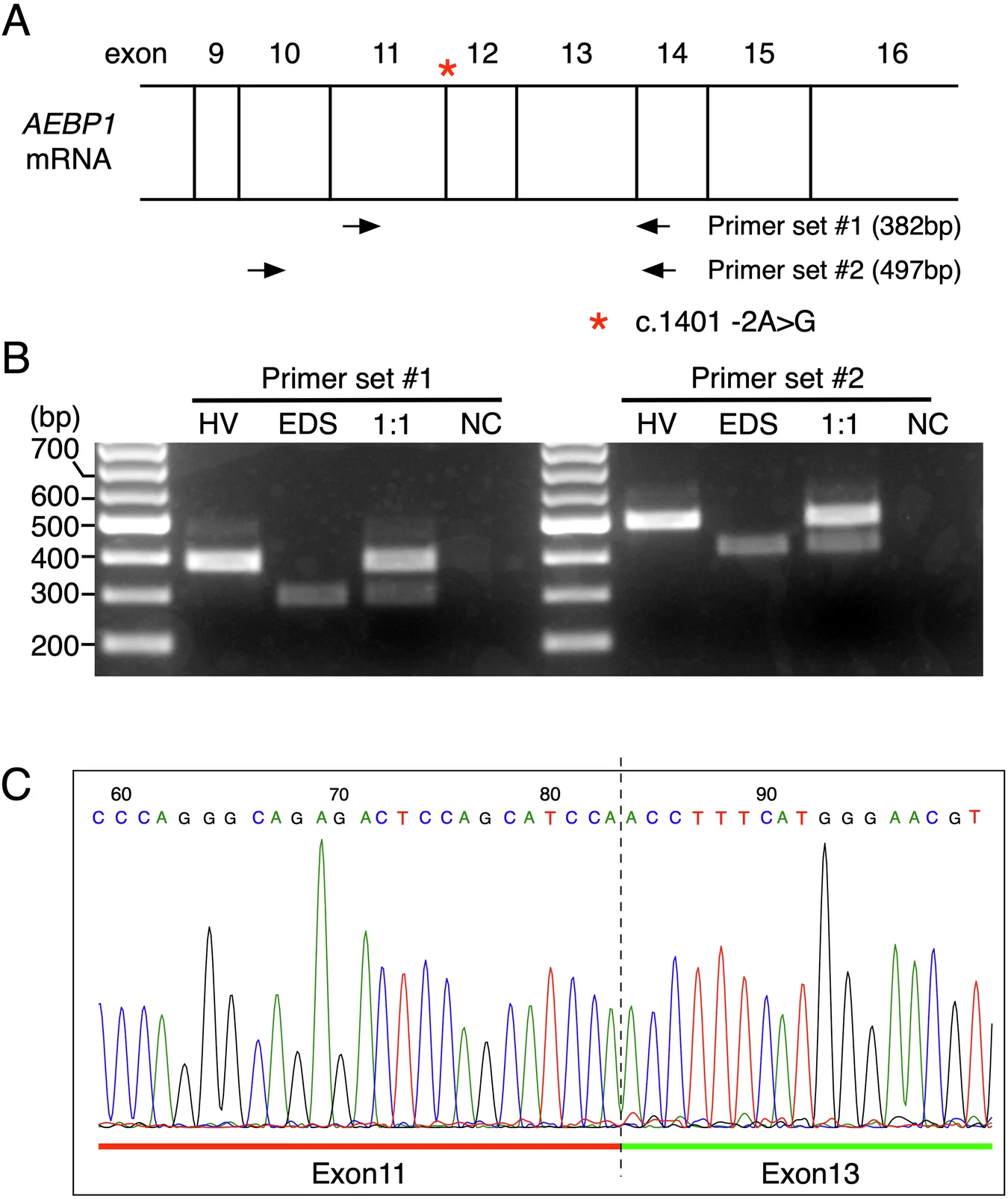
A novel *AEBP1*:c.1401-2 A > G variant induces exon 12 skipping, resulting in a frameshift. **A** Primer design for reverse transcription-polymerase chain reaction (RT-PCR). Also see prediction of splicing alterations and primers described in Fig. 2 and Supplementary Table 1. **B** RT-PCR from blood from the clEDS2 patient (EDS), compared to one from a healthy volunteer (HV). To clarify the difference in amplicon size between HV and EDS, 1:1 mixture of RT-PCR products from HV and EDS was electrophoresed. The amplicon size is approximately 80 base pairs shorter. **C** Sanger sequence from amplicon from EDS, which reveals exon 12 skipping resulting in frameshift

The detected variant was not registered in population allele frequency databases, including gnomAD [15] and jMorp [16]. To investigate the mechanism of functional deficiency of *AEBP1* due to the novel variant in *AEBP1*, we first performed in silico analysis using SpliceAI, showing that a splice acceptor signal in intron 11 disappeared with the variant. Instead, a predicted alternative splice acceptor site appeared 36 base pair (bp) downstream of the canonical acceptor site (Fig. 2A). Based on this in silico analysis, we presumed two splicing alterations: the first is 36 bp in-frame deletion (p.Asp468_Ser479del) resulting in huge conformational alteration in the discoidin domain (Fig. 2B, C); the second is exon 12 skipping leading to 85 bp stop-gain deletion (p.His467Glnfs*14, PVS1, PM2_supporting) (Fig. 2D). To validate a predicted splicing alteration predominantly caused by the variant, we performed RT-PCR followed by Sanger sequencing. To that end, we designed two independent primer sets between exon 11 and 13 (Fig. 3A). By RT-PCR, a shorter amplicon was obtained from the patient, compared to one from a healthy volunteer (Fig. 3B). Then, the shorter amplicon from the patient was examined by Sanger sequencing. Finally, exon 12 was found to be skipped with exon 11 directly connected to exon 13 (Fig. 3C).

### Histopathological findings of the colon

**Fig. 4:**
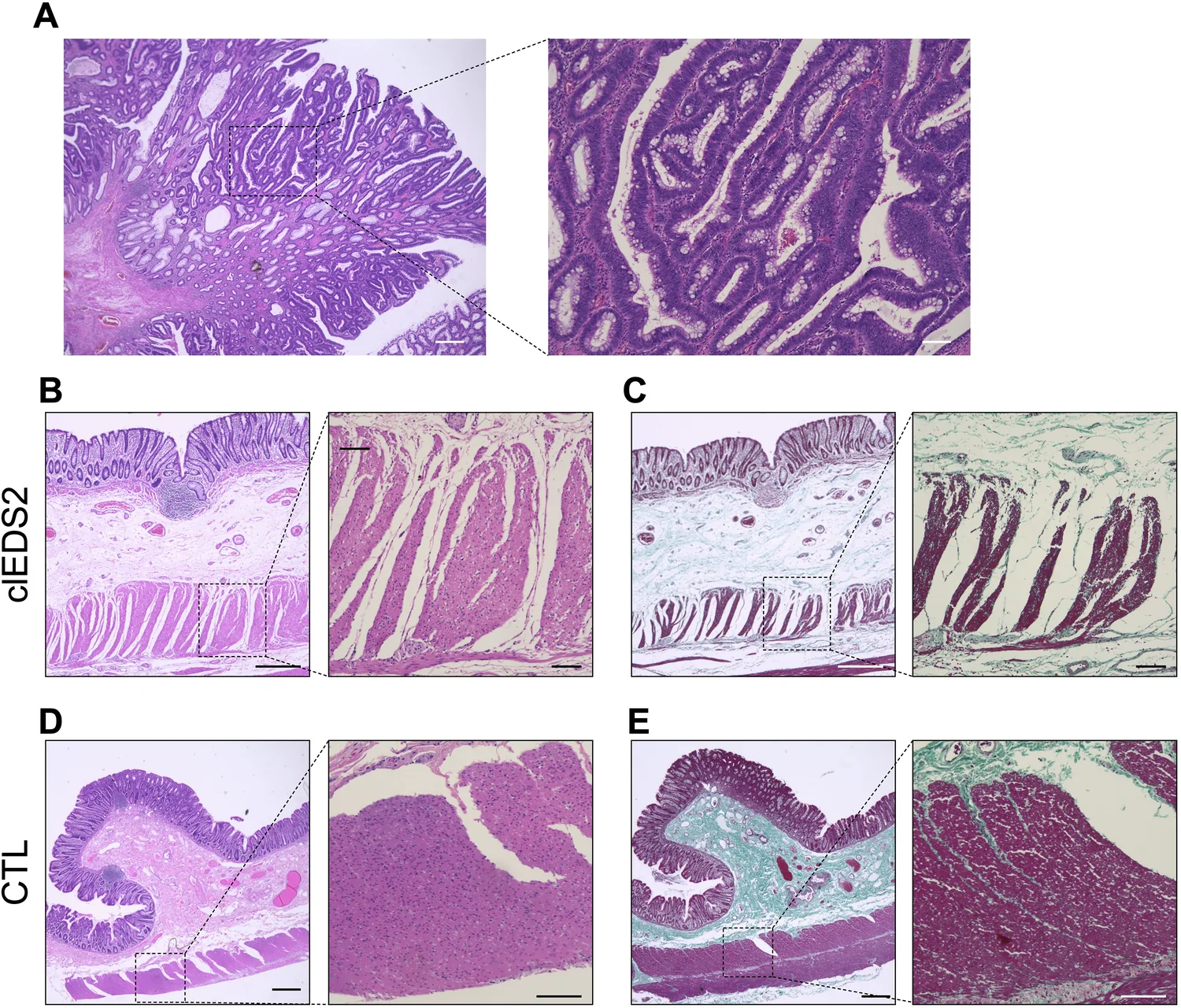
Pathological findings of the colon upon the proctosigmoidectomy and the emergency laparotomy with partial colectomy for colonic perforation. **A** Hematoxylin and eosin (H&E) staining for rectosigmoid cancer at the age of 54 indicates well differentiated adenocarcinoma with tubular adenoma. H&E (**B**) and Masson’s trichrome (MT) (**C**) staining for adjacent normal colonic tissue from clEDS2 patient. H&E (**D**) and MT (**E**) staining for adjacent normal colonic tissue from counterpart control patient (CTL) who underwent rectosigmoidectomy during a similar period. High-magnification views correspond to the areas indicated by dashed boxes. Scale bar: 500 µm and 100 µm for low and high magnification, respectively

Histopathological examination of the rectosigmoid tumor demonstrated a well-differentiated adenocarcinoma with tubular adenoma, showing no unusual histological features beyond those typically observed in sporadic colorectal cancer (Fig. 4A). To assess gastrointestinal involvement at the histopathological level, the adjacent non-neoplastic colonic tissue was evaluated in comparison with a counterpart control obtained from a patient who underwent rectosigmoidectomy during a similar period. H&E and MT staining of adjacent non-neoplastic colonic tissue in the patient with clEDS2 showed largely preserved tissue architecture, comparable to that observed in the counterpart control specimen. In colonic tissue obtained in proximity to the perforation that occurred two months after proctosigmoidectomy, mild widening of the intermuscular spaces was observed, accompanied by subtly attenuated collagen fibers (Fig. 4B–E). It remains unclear whether these histopathological findings reflect secondary changes related to altered mechanical stress of the bowel wall due to collagen dysfunction, or whether they represent primary structural alterations intrinsic to *AEBP1*-related clEDS2. No other overt structural abnormalities relative to the control tissue, including transmural disruption, inflammatory changes, or marked fibrosis, were identified in the bowel wall.

## Discussion

In this study, we clinically and genetically characterized a patient with clEDS2 caused by a novel *AEBP1* splice-site variant. During routine long-term oncological surveillance following rectal cancer surgery, repeated gastrointestinal endoscopic procedures were performed over a 20-year period without procedure-related bowel perforation. While *AEBP1*-related clEDS2 is an extremely rare autosomal recessive connective tissue disorder, accumulating evidence has highlighted marked clinical heterogeneity, particularly with respect to cardiovascular and gastrointestinal involvement [2, 12]. The present observation expands the molecular and phenotypic spectrum of clEDS2 by demonstrating a previously unreported splicing alteration leading to exon skipping and frameshift, together with rare longitudinal documentation of spontaneous bowel perforation.

From a molecular genetic perspective, the identified *AEBP1* variant (c.1401-2 A > G) represents a novel splice-site alteration affecting the canonical acceptor site of intron 11. In silico splice prediction suggested disruption of normal splicing at the exon–intron boundary, prompting experimental validation at the transcript level. RT–PCR followed by Sanger sequencing demonstrated complete skipping of exon 12, resulting in a frameshift and premature termination of the AEBP1 protein. Biallelic loss-of-function variants in *AEBP1* have been established as the molecular basis of clEDS2, with previously reported pathogenic variants including nonsense, frameshift, and splice-altering mutations distributed across functional domains of the protein [5, 13, 14]. The present splice-site variant further expands the mutational spectrum of *AEBP1* by providing direct experimental evidence of exon skipping leading to predicted loss of function. These findings are consistent with the established pathogenic mechanism of *AEBP1*-related clEDS2, which involves functional deficiency of AEBP1, and underscore the importance of transcript-level analyses when evaluating intronic or splice-site variants in rare connective tissue disorders.

Gastrointestinal involvement in EDS varies substantially by subtype, with spontaneous bowel perforation being a well-recognized and life-threatening complication predominantly associated with vascular EDS [2]. In contrast, such events have been only rarely documented in clEDS2, and detailed pathological or longitudinal clinical information has been limited [12]. To date, only a single case of bowel rupture in *AEBP1*-related clEDS2 has been reported **[**5], in which diverticulosis was considered a possible contributing factor; however, histopathological findings of the perforation site were not described, precluding direct comparison with the present observation.

In the present observation, the patient experienced two bowel perforation events over a 20-year period: postoperative perforation in the sigmoid colon shortly after rectal cancer surgery, and spontaneous small intestinal perforation nearly 20 years later without an identifiable precipitating factor. The relatively advanced age at perforation in the present case may suggest that gastrointestinal complications in *AEBP1*-related clEDS2 can occur later than those typically reported in vascular EDS [17], although additional cases are required to clarify this point.

Histopathological examination of the affected colonic tissue demonstrated only subtle structural alterations, characterized by mild widening of the intermuscular spaces with slightly attenuated collagen fibers, and no additional overt abnormalities, including transmural disruption, inflammatory changes, or marked fibrosis, were identified in the bowel wall.

Within the context of the extremely limited number of reported cases, of which 15 affected individuals from 13 families have been described since the initial identification of clEDS2 in 2016 [4], this observation represents the 16th genetically confirmed case of *AEBP1*-related clEDS2 and is distinguished by two notable features: the identification of a previously unreported splice-site variant with experimentally validated exon skipping, and longitudinal clinical documentation of spontaneous bowel perforation over a 20-year period following rectal cancer surgery. Taken together, these findings emphasize the clinical heterogeneity of clEDS2 and suggest that gastrointestinal complications, while uncommon, should be considered as part of the phenotypic continuum associated with *AEBP1* deficiency.

Gastrointestinal endoscopic procedures are often approached with caution in patients with EDS because of the well-documented risk of procedure-related bowel perforation in vEDS [2]. Indeed, previous studies have reported an estimated incidence of endoscopy-related bowel perforation of approximately 9.4% in vascular EDS, compared with less than 1% in classical and hypermobile EDS [18]. However, data regarding the safety of endoscopic surveillance in clEDS2 remain extremely limited. In the present observation, repeated upper and lower gastrointestinal endoscopic examinations were performed over a 20-year period as part of routine oncological surveillance, without procedure-related bowel perforation. Although no general conclusions can be drawn from a single observation, this longitudinal experience suggests that gastrointestinal complications in *AEBP1*-related clEDS2 may differ in clinical context from those typically observed in vEDS. These findings highlight the importance of precise EDS subtyping and individualized clinical judgment when planning gastrointestinal surveillance. Further accumulation of cases with detailed procedural histories will be essential to better define the gastrointestinal risk profile associated with clEDS2. Because these observations are derived from a single case, the histopathological findings should be interpreted cautiously. Nevertheless, they highlight aspects of gastrointestinal involvement that warrant further investigation as additional cases of *AEBP1*-related clEDS2 are accumulated.

Taken together, this case illustrates that gastrointestinal involvement in *AEBP1*-related clEDS2 may include both postoperative and spontaneous bowel perforation, underscoring the importance of subtype-specific risk assessment in the clinical management of EDS.

## Supplementary information

Supplementary Table 1

ICMJE disclosure form

## Data Availability

The data of the current study are not openly available due to ethical considerations and are available from the corresponding author upon reasonable request.
